# Workforce Representation and Service in Shortage Areas: Outcomes of a HBCU College of Dentistry, Class 2004–2023

**DOI:** 10.1002/jdd.70105

**Published:** 2025-11-12

**Authors:** Marzia Mustamand, Minxuan Lan, Esther Childers, Cheryl Fryer, Kingsley Kanjin, Emma Ta‐Zhou, Najah Abduh, Whittley Deleveaux, Raheem Pierre, Gail Cherry‐Peppers, Reginald Salter, Deborah Nixon‐Willis, Tanya Greenfield, Dexter Woods, Andrea Jackson, Xinbin Gu

**Affiliations:** ^1^ Howard University College of Dentistry Washington District of Columbia USA; ^2^ Department of Geography and Planning The University of Toledo Toledo Ohio USA; ^3^ Walt Whitman High School Bethesda Maryland USA

**Keywords:** dental clinic, dental education, health disparities, Medicaid, underserved areas, workforce representation

## Abstract

**Purpose:**

This study examines Howard University College of Dentistry's (HUCD) contributions to dental education, focusing on its role in strengthening workforce representation, expanding access to care in dental health professional shortage areas (DHPSAs) and medically underserved areas (MUAs), and addressing persistent oral health disparities.

**Methods:**

A retrospective analysis was conducted of 1473 HUCD graduates from the Classes of 2004–2023, using data verified through publicly available sources. Variables included gender, race/ethnicity, and practice location. Geographic Information System (GIS) mapping identified practices within DHPSAs and MUAs, and the two‐step floating catchment area (2SFCA) method was used to calculate accessibility scores for census tracts in Washington, DC.

**Results:**

Among 1292 graduates with complete data, 59% were female and 50% identified as African American. On average, 68.4% of graduates practiced in DHPSAs or MUAs, with higher levels of service in communities with larger African American (19.98%) and Hispanic/Latino (20.10%) populations. GIS analysis showed concentrations of HUCD graduates in the Washington, DC, Beltway and Texas. The 2SFCA mapping revealed that Wards 5, 7, and 8, areas with the largest African American populations in Washington, DC, experience the most significant access challenges, and the HUCD's clinic primarily serve patients from these areas.

**Conclusion:**

HUCD has demonstrated success in preparing graduates to provide care in underserved communities, advancing its mission to improve oral health outcomes. These findings highlight the importance of continued investment in educational models that strengthen the workforce and expand access to care.

## 1 Introduction

Oral health represents an essential component of an individual's overall health and wellness [1]. The oral structures play a key role in maintaining human health and social function [1]. It is the gateway through which we receive nutrition, outwardly communicate, and display emotional expression covering physical, mental, social, and economic aspects of an individual's life [1]. Poor oral health has been tied to systemic diseases that affect overall health as well [2, 3]. Research suggests that periodontal treatment leads to an improvement in glycemic control in type 2 diabetic patients [2, 3].

Many factors and social determinants contribute to inequalities across the nation. Barriers such as limited financial resources, inadequate transportation, and a poor understanding of how oral health impacts overall health are common. The 2021 Surgeon General report on oral health emphasized that there are “deep disparities in the experience of disease by different population groups and the systemic inequities in access to care,” which mainly affect those from low‐income backgrounds and racially and ethnically marginalized groups [1]. People with lower incomes often have limited access to dental insurance, and Medicaid generally provides minimal dental benefits [1, 4]. Additionally, this inequity in dental care access varies between and within states and worsens due to the shortage of dental professionals in underserved areas [5, 6].

Race and ethnicity have been established as crucial stratification factors when examining oral health disparities [7]. This is due to the unequal distribution of dental healthcare, as well as socioeconomic disparities, across different racial and ethnic populations [7]. The concept of racial and ethnic concordance has been supported and established as a predictor, indicating that patients are more likely to seek out and prefer physicians and medical professionals of their race or ethnicity [8]. However, there has not been comprehensive, systematic research on how patients perceive dentists from their race or ethnic background [9]. Conversely, studies have demonstrated that minority dentists tend to care for significantly more patients of their own race compared to the demographic composition of the local population [10]. Such findings suggest that increasing representation within the dental workforce may enhance access and promote racially concordant care [10]. Those authors believe the initiative to foster a broad dental workforce can begin by enrolling a variety of culturally sensitive and competent students in dental colleges [11].

Decades of evidence show that higher education institutions, including dental colleges, have higher enrollment of socially dominant racial and ethnic groups [12]. This has been described as a pathway through which oral health disparities have persisted, and it is through the lack of representation in the dental workforce that these inequities in oral care have been sustained [11]. Furthermore, oral health disparities cannot be fully addressed without increasing Medicaid dental providers nationally. Medicaid‐accepting dentists in Iowa have reported having positive attitudes towards the Medicaid administration and tend to feel more altruistic than their non‐Medicaid counterparts [13]. Underrepresented minority (URM) dental students express greater altruism and an eagerness to accept Medicaid patients and serve shortage areas [10, 11, 14]. URM‐graduated dentists were also three times more likely to work in a public‐sector setting compared to the traditional dentistry environment [10]. Cultural competence and sensitivity training in dental school can facilitate an increase in intentions to serve patients from racially/ethnically different backgrounds [10]. Also, the recruitment of URM students at dental schools can aid in an increase in Medicaid‐accepting dental providers [10, 14]. Ultimately, those authors believe that dental colleges can improve oral health outcomes through promoting cultural competence and altruism.

Howard University College of Dentistry (HUCD), one of only two HBCU dental schools, has long been recognized for its excellence and service. It stands as a leading contributor to the education and advancement of African American dental professionals nationwide. Guided by its mission, the College is dedicated to improving the quality of oral healthcare across communities at local, national, and global levels. The purpose of this study is to evaluate the outcomes of HUCD graduates over the past 20 years, with particular attention to their practice locations and patient populations, and to assess whether they provide oral healthcare to medically and dentally underserved communities nationwide. In addition, this study analyzes the demographics and residential areas of HUCD's dental patients and conducts a comprehensive assessment of the economic and healthcare characteristics of these regions.

## 2 Methods

### 2.1 Data Compilation Procedure

We conducted a thorough analysis of the graduates from HUCD, spanning the classes of 2004 to 2023. This analysis uses National Provider Identifier (NPI), Google, LinkedIn, and the Health Resources and Services Administration (HRSA)’s shortage designation platform. Dentistry locations were collected online, input into a database, and then coded by zip code and region. As it stands, from 2004 to 2023, about 1473 students graduated from the College of Dentistry, and the number of active dentists is 1292. The dataset we have accumulated includes gender, race, ethnicity, primary and secondary practice locations, as well as confirmation of whether these locations are in dental shortage and medically underserved areas (MUA/P). For FY2023–2024, HUCD dental clinical data collection was limited to patients’ age, gender, and residential ZIP codes, without any personally identifiable information. The study was reviewed and granted exempt status by the Howard University Institutional Review Board (IRB‐2021‐0056, approval date August 3, 2021) in accordance with 45 CFR 46.101(b)(2).

### 2.2 Data Collection Platforms

*Provider Identity and Practice Information*: The NPI Registry (https://npiregistry.cms.hhs.gov/) was used to verify provider identity, as well as clinical practice location and address. Supplemental biographical and professional details were collected from online platforms such as Google and LinkedIn.

*Underserved Area Classification*: Practice locations were assessed against federal designations of MUAs and Health Professional Shortage Areas (HPSAs) using data from the HRSA (https://data.hrsa.gov/).

### 2.3 GIS Mapping and 2SFCA

Geographic Information System (GIS) mapping was employed to identify clusters of graduate practice locations, evaluate proximity to underserved areas, and visualize spatial distribution patterns. To further analyze the Washington, DC region, the two‐step floating catchment area (2SFCA) method was used to assess spatial accessibility to dental care by calculating the ratio of dental facilities to population within census tracts. To represent population distribution, centroid points were generated for each census tract and assigned population values corresponding to their respective tracts, serving as proxies for geographic population centers. An origin‐destination (OD) matrix was then created to calculate the distance between each dental facility and population centroid. This distance matrix served as the foundation for defining interaction zones (or catchment areas) used in the 2SFCA analysis. The tool computed the ratio of dental facilities to population within each catchment and normalized the results to represent facilities per 1000 people. The resulting accessibility scores reflect the spatial accessibility of dental care availability: higher scores indicate better access, while lower scores highlight potential service gaps.

### 2.4 Comprehensive Data Analysis

Practice sites were assessed for inclusion within HRSA‐designated MUAs and HPSAs. Using this software, we were able to overlay the primary and secondary practice locations of Howard dental graduates on a map of the United States to identify where our graduates practice by zip code. The racial and ethnic composition of the areas in which our dental graduates practice was analyzed to evaluate potential concordance between providers and the communities they serve. Dental shortage and MUA/P locations were extracted by zip code and address to determine the percentage of HUCD's graduates serving in these designated areas. These zip codes were then overlaid on a map to visualize the geographic distribution and areas of concentration. The percentage was derived by quantifying the number of active dentists from a given graduating cohort practicing in dental health professional shortage areas (DHPSAs) or MUAs and dividing this by the total number of graduates in that cohort.

## 3 Results

### 3.1 Study Sample

Between the years 2004 and 2023, a total of 1473 dental students graduated from HUCD. Comprehensive data was successfully collected and confirmed for 1292 graduates, representing 87.7% of the entire cohort. Graduate identification and practice details were verified using the NPI database, supplemented by publicly available information. Individuals excluded from the study included those lacking available NPI information, unverifiable practice details, relocation outside the United States, or death.

### 3.2 Graduate Demographic and Representation

**FIGURE 1:**
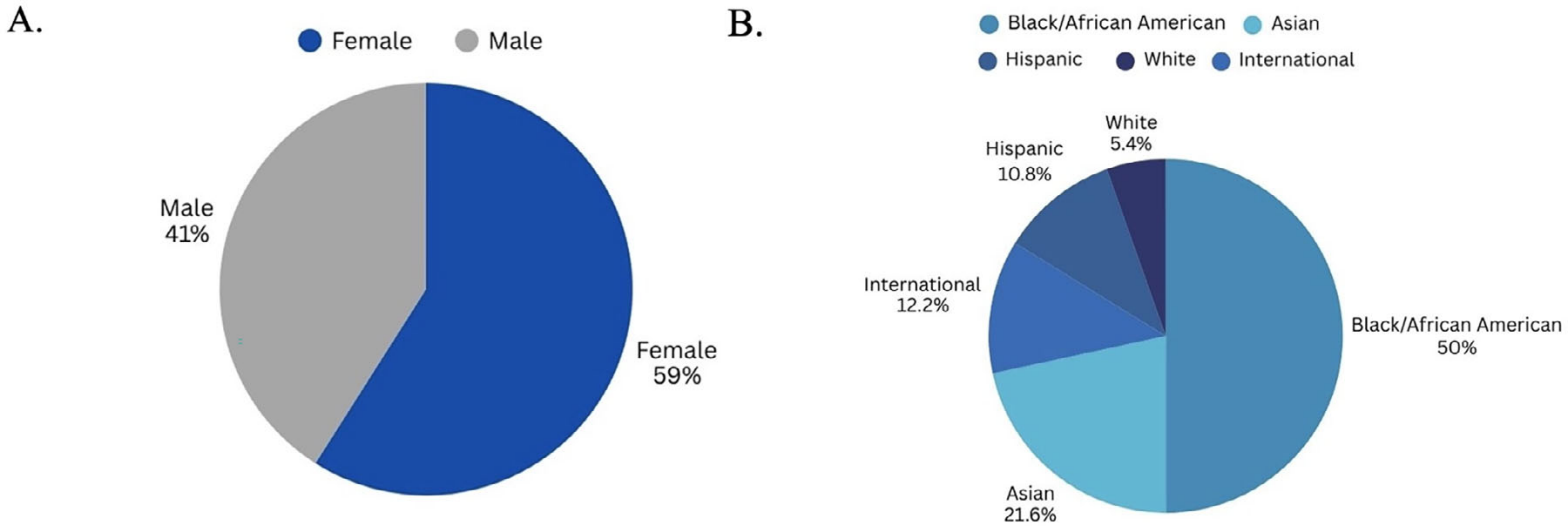
Gender and racial/ethnic composition of HUCD students (2004–2023). (A) Female‐to‐male enrollment ratio at Howard University College of Dentistry over a 20‐year period. (B) Racial and ethnic makeup of HUCD students during the same period.

Among the active 1292 graduates from HUCD, 59% identify themselves as female and 41% identify themselves as male, which is in line with the national trends that indicate that the percentage of female dental school graduates is increasing (Figure 1A). The class years of 2019–2023 were selected to serve as a representative subset of HUCD's racial and ethnic composition, given that these cohorts provide the most complete dataset. As shown in Figure 1B, HUCD racial and ethnic composition for this subset is the following: Black or African American (50%), Asian (21.6%), International (12.2%), White (10.8%), and Hispanic (5.4%).

### 3.3 Overall Graduate Placement

**FIGURE 2:**
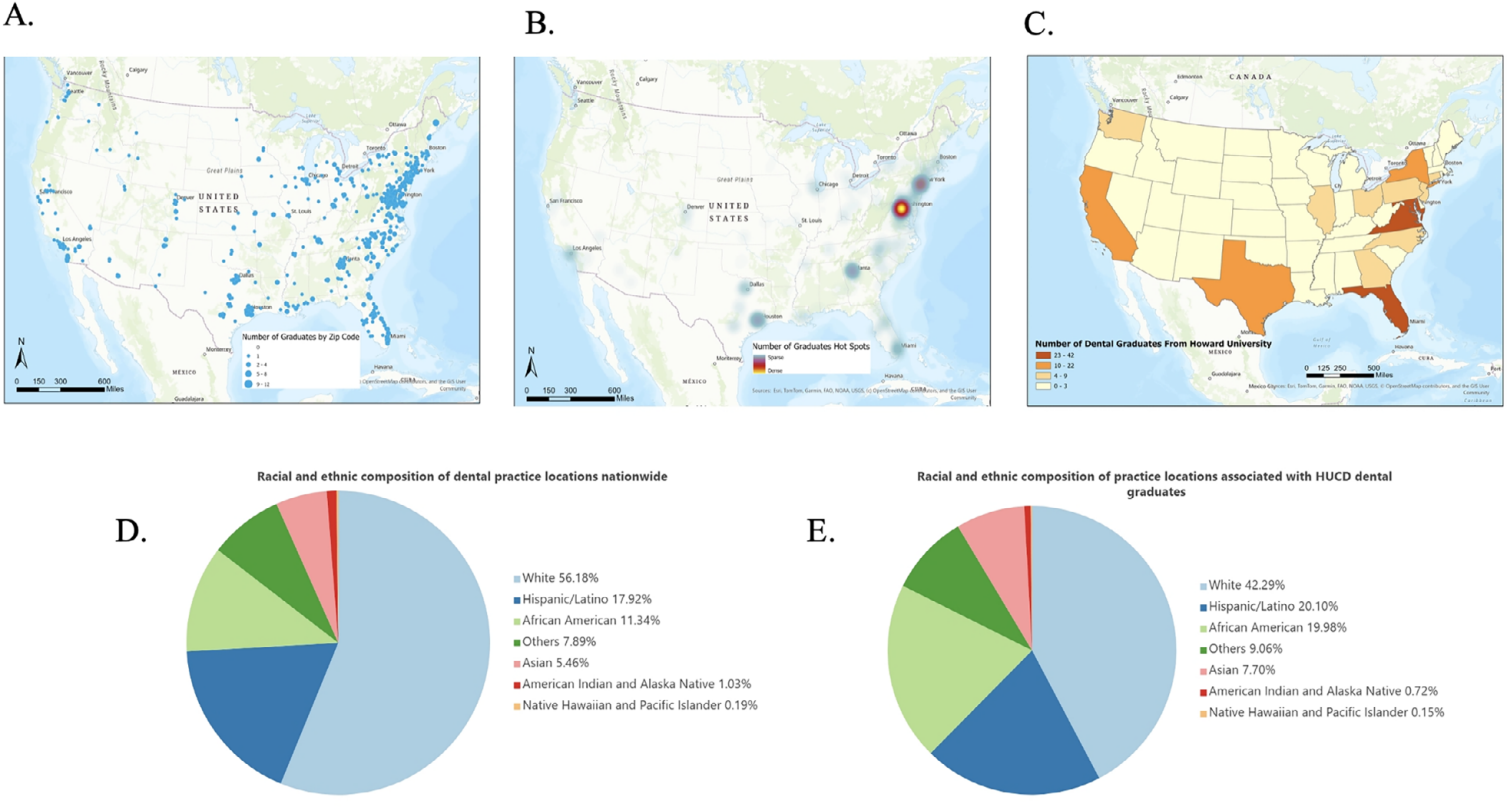
Geographic distribution and demographic composition of HUCD dental graduates (Classes 2004–2023). (A) GIS point count of HUCD dental graduates providing oral healthcare by ZIP code. (B) Hotspot analysis of graduate practice locations. (C) Heat map of practice distribution by state. (D) Racial and ethnic composition of dental practice locations nationwide. (E) Racial and ethnic composition of practice locations associated with HUCD dental graduates.

Graduates from HUCD practice mainly within the East Coast of the United States (Figure 2A,B). The highest areas of concentration are Washington, DC, Maryland, and Virginia regions (Figure 2A,B). Smaller clusters and sparse hotspots exist in New York, Florida, Georgia, Texas, and California (Figure 2A,B). Numerous graduates serve urban regions characterized by greater minority representation. Compared to dental practice locations across the country, Howard University's dental graduates work in areas with larger African American (19.98%) and Hispanic/Latino (20.10%) populations (Figure 2C,D).

### 3.4 Practice in Underserved Areas (MUAs/DHPSAs)

**FIGURE 3:**
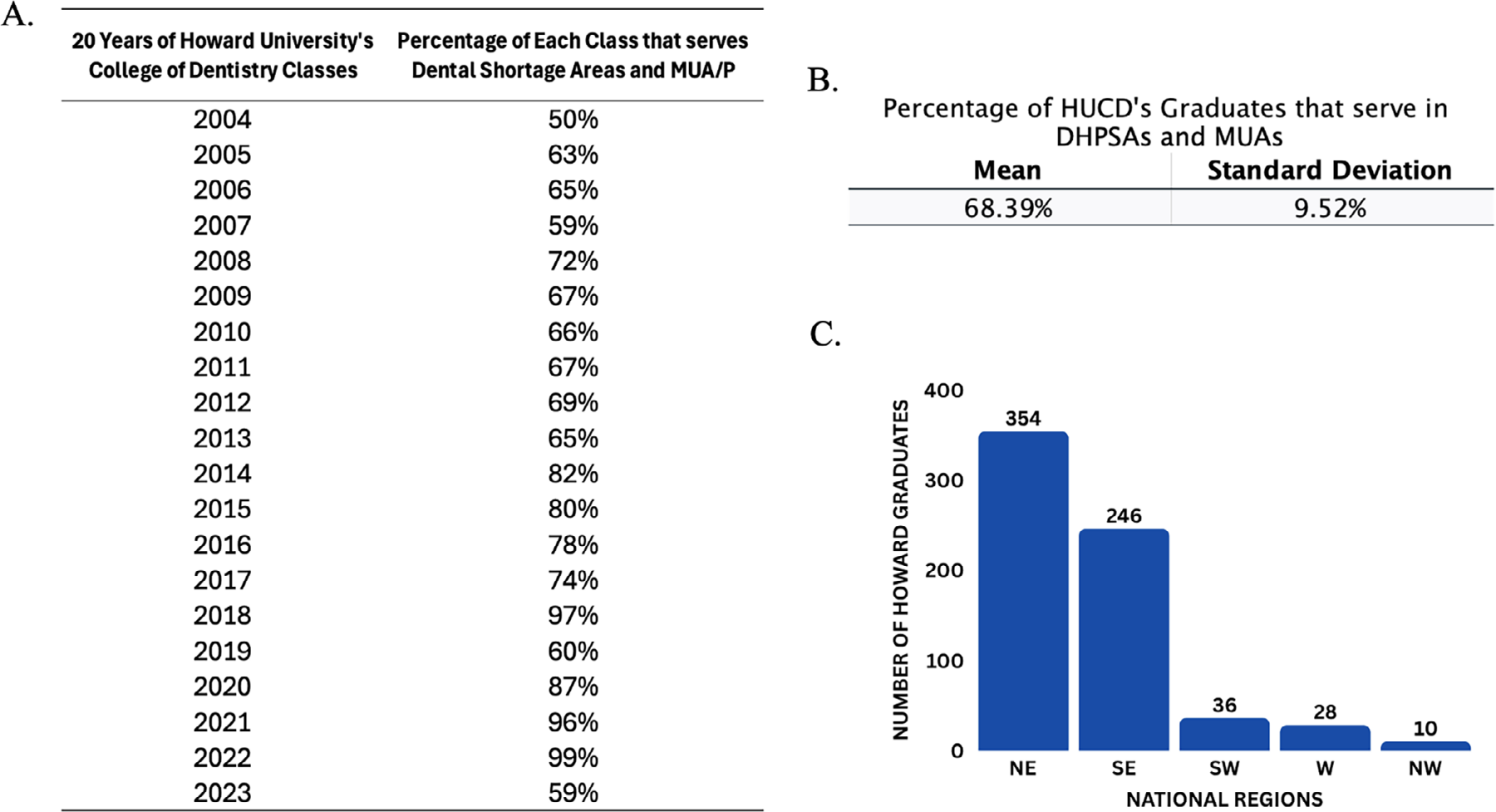
HUCD graduates serving in DHPSAs and MUAs. (A) Percentage of each HUCD graduating class (2004–2023) serving in DHPSAs and MUAs. (B) Mean percentage and standard deviation of HUCD graduates serving in DHPSAs and MUAs across the 20‐year period. (C) Regional distribution of HUCD graduates serving in DHPSAs and MUAs, shown by national regions (NE, SE, SW, W, NW).

Geospatial analysis combined with HRSA data revealed that at least 50% (mean = 68.39%) of each class (Figure 3A), spanning from 2004 to 2023, were providing oral healthcare in federally designated MUAs and DHPSAs. It should be noted that there is a sharp increase in graduates serving in DHPSAs and MUAs within the class years of 2018, 2021, and 2022. These class years can be acknowledged as outliers and are removed from the mean (68.39%) and standard deviation (9.52%) to provide an accurate representation of the data (Figure 3B). These results indicate that a significant proportion of the graduates are practicing in dental shortage and medically underserved areas, aligning with the program's mission to address oral health disparities.

**FIGURE 4:**
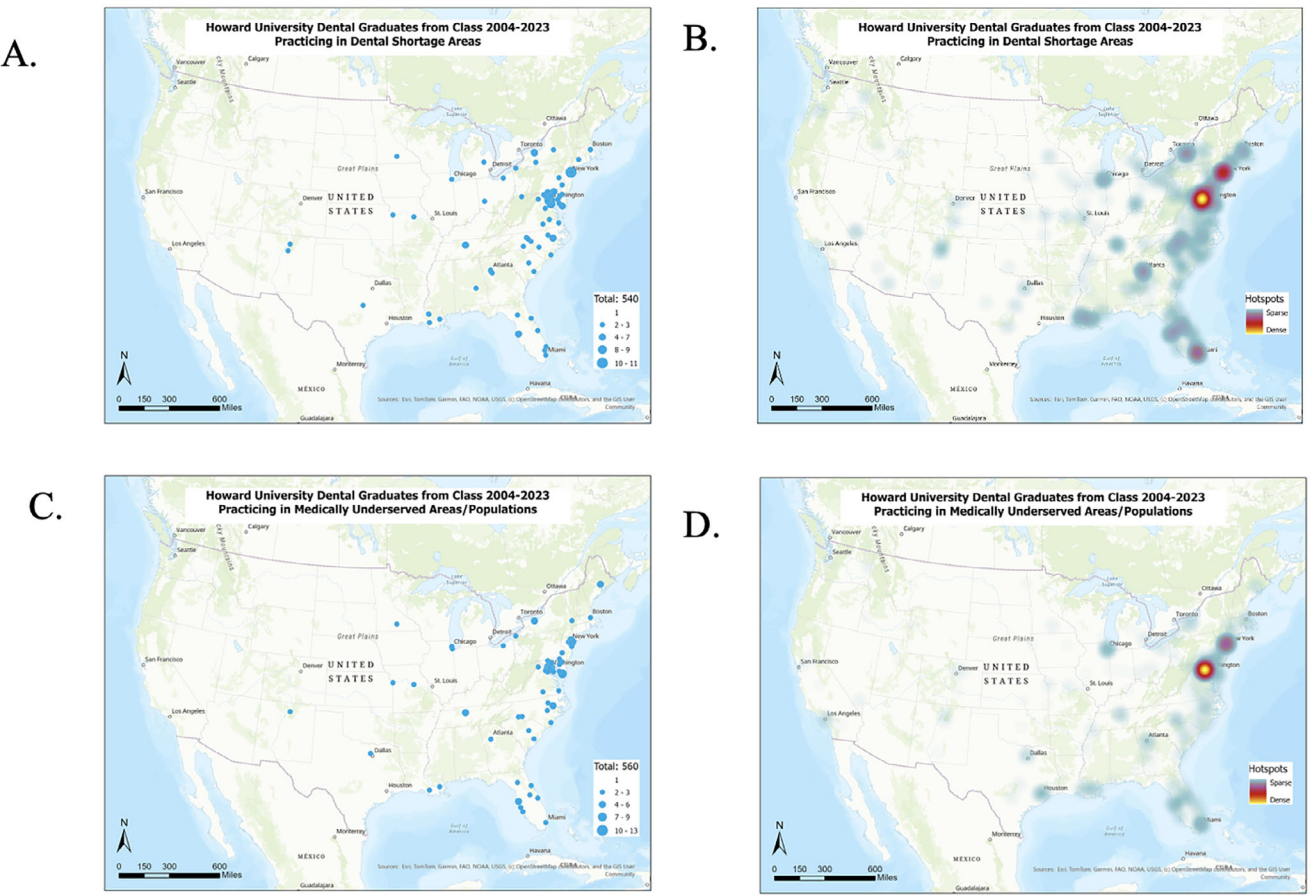
Geographic distribution of HUCD graduates serving in dental shortage and medically underserved areas (Classes 2004–2023). (A) GIS point mapping of HUCD graduates practicing in federally designated dental shortage areas, represented as counts by ZIP code. (B) Hotspot analysis of HUCD graduate practice locations within dental shortage areas. (C) GIS point mapping of HUCD graduates practicing in medically underserved areas, represented as counts by ZIP code. (D) Hotspot analysis of HUCD graduate practice locations within medically underserved areas.

Graduates from HUCD who practice in DHPSAs and MUAs are geographically distributed across five national regions of the United States (Figure 3C). The highest concentration areas are the Northeastern (*n *= 354) and Southeastern (*n *= 246) regions, while there is some sparse placement within the Southwestern (*n *= 36), Western (*n *= 28), and Northwestern (*n *= 10) regions of the United States (Figure 3C). When examined separately, the DHPSA maps (Figure 4A,B) and MUA maps (Figure 4C,D) both reveal that dense clusters are present Washington, DC, Maryland, Virginia region, while moderately dense clusters are present in the New York area. These findings suggest that our dental graduates have consistently delivered oral healthcare services to underserved communities across the Northeastern and Southeastern regions of the United States over the past 20 years.

### 3.5 HUCD Dental Clinical and Community‐Based Training Outcomes

Clinical competency milestones are evaluated alongside patient volume and the number of clinical encounters per student, with a specific emphasis on patient age groups and gender (Figure 6D,E). Consistent with its mission, HUCD expects students to attain academic and clinical competencies specific to dental education, patient care, research, and community service, thereby equipping them to deliver effective oral healthcare to diverse and underserved populations. Collectively, these metrics ensure that students receive comprehensive, real‐world training to effectively address the evolving demands of patient care. Within FY 2023–2024 alone, there have been 27,913 patient visits at the HUCD dental clinic.

**FIGURE 5:**
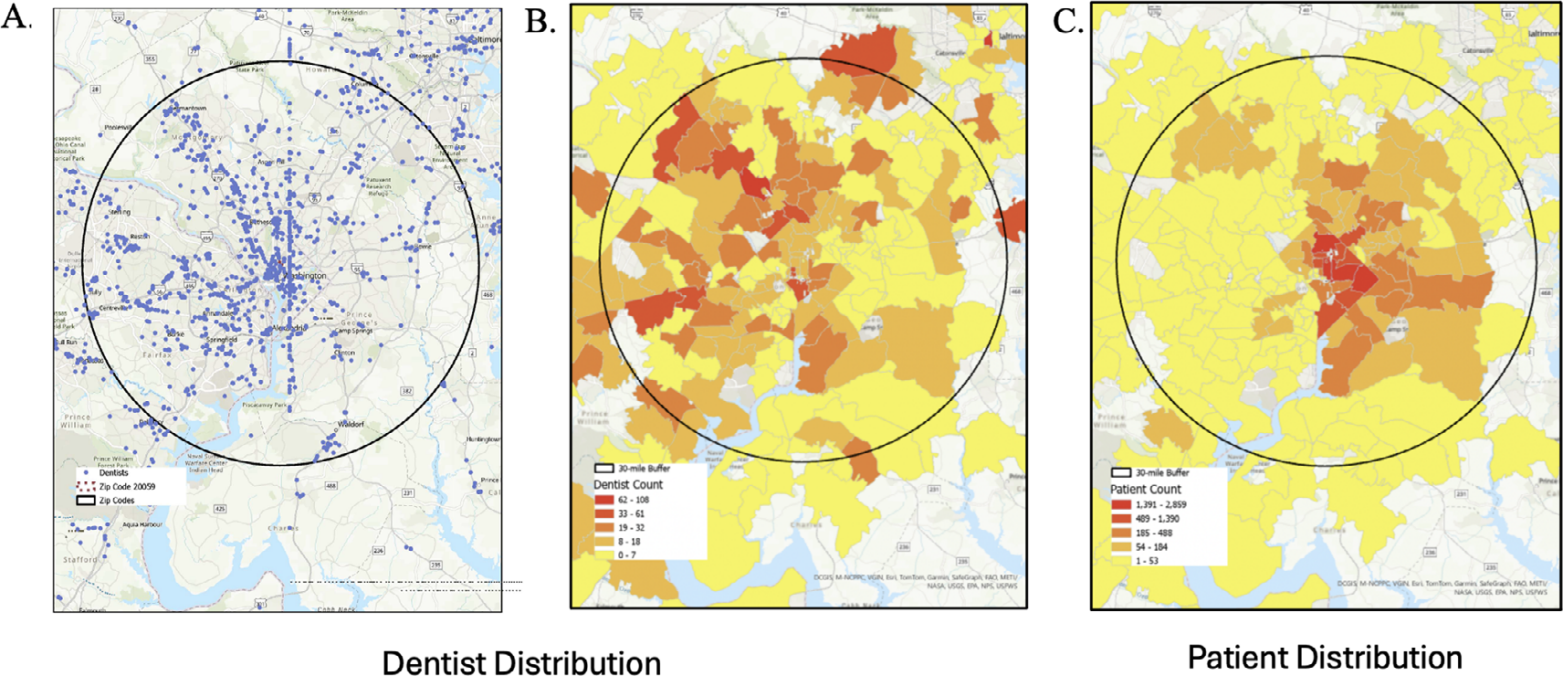
Distribution of dental providers and patients within 30 miles of HUCD. (A) Geographic locations of dental care providers within a 30‐mile radius of HUCD. (B) Heatmap distribution of dental providers across the catchment area. (C) Residential distribution of HUCD dental patients within the 30‐mile service area.

Figure 5 illustrates the regional distribution of dental resources and patient origins, with HUCD located at the center of a 30‐mile catchment area. Figure 5A,B maps the dental clinics across the region, showing a higher concentration of practice sites in central and western urban areas, with density tapering off toward the east and outer periphery. Figure 5C depicts the residential locations of HUCD patients, indicating that the College draws a substantial share of its patients from within this 30‐mile radius, particularly from the eastern areas. Together, these maps highlight HUCD's role as a central safety‐net institution, serving both underserved urban wards and surrounding suburban communities.

**FIGURE 6:**
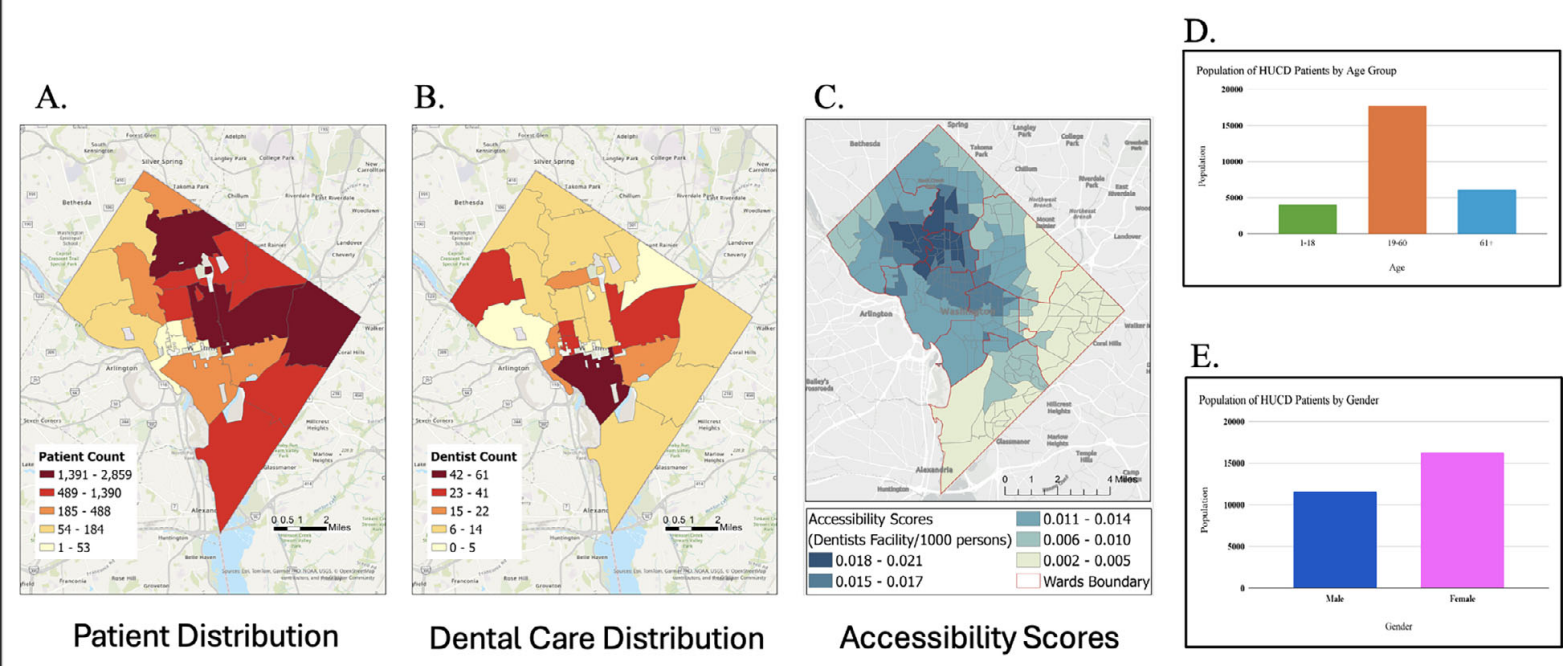
HUCD patient distribution, provider availability, and accessibility in Washington, D.C. Area. (A) Geographic distribution of HUCD patients by ZIP code. (B) Distribution of practicing dentists across the same area. (C) Accessibility scores for dental care services were calculated using the two‐step floating catchment area (2SFCA) method. (D) HUCD patient population by age group. (E) HUCD patient population by gender.

Figure 6 examines the Washington, DC metropolitan area by integrating patient population data with accessibility modeling. Figure 6A shows the distribution of HUCD patients across the city's wards. Although patients come from all wards, higher concentrations are seen in centrally located areas. Figure 6B presents a map showing the distribution of dental clinics throughout the region. Figure 6C reports accessibility scores generated using the 2SFCA method, revealing marked variation in dental care availability relative to population need. Notably, Wards 6, 7, and 8 exhibit significantly lower accessibility scores, reflecting limited access to dental services despite demonstrated patient demand. This highlights a misalignment between where patients live and where services are available, underscoring persistent inequities in oral health access across the city. The majority of HUCD patients are between 19 and 60 years of age and identify as female (Figure 6D,E). This high patient volume provides HUCD students with valuable opportunities to develop the skills needed to deliver comprehensive care across a variety of demographic groups.

In addition, HUCD's curriculum places strong emphasis on community service, integrating outreach into student training to prepare future dentists to address oral health disparities through direct, hands‐on experience. Students provide care in diverse settings, including the on‐campus clinic that offers reduced‐cost services and accepts Medicaid, as well as HUCD students joined 236 professional schools from 35 states in delivering urgently needed oral health care through the Remote Area Medical (RAM) organization. Collectively, Howard dental students have contributed 19,312.25 volunteer hours, the equivalent of 804.7 days, or 2.2 years, of continuous care. HUCD maintains internal institutional logs documenting students’ volunteer service hours, reflecting the College's mission to incorporate community engagement into all aspects of dental education. The college considers an average of 20–30 h of service annually as a general benchmark for student participation in community‐based activities. According to our internal institutional logs, about 20% of students exceed a total of 200 h by graduation. The recorded hours represent meaningful opportunities for students to engage with the community, apply classroom knowledge in real‐world settings, and develop a professional identity rooted in service. Although there are no national benchmarks for direct comparison, the documentation of student contributions highlights HUCD's ongoing commitment to service as a fundamental professional skill.

## 4 Discussion

Evidence from this study demonstrates that HUCD is making measurable progress in reducing oral health disparities while developing a healthcare workforce that reflects underserved communities. Between 2004 and 2023, an average of 68.39% of College of Dentistry graduates have provided oral healthcare in federally designated dental shortage and medically underserved areas across the United States. Notably, 50% of HUCD graduates identify as Black or African American, a stark contrast to their 3.8% representation in the national dental workforce. Through participation in RAM experiences, HUCD students deliver both tangible health benefits to underserved populations and gain invaluable educational opportunities. These service‐learning experiences enhance clinical skills while embodying the school's mission to train socially conscious, community‐focused dental professionals. HUCD also ranks among the nation's professional schools with the highest volunteer hours, underscoring its leadership and unwavering commitment to service. These findings expand on prior evidence of HUCD's commitment to preparing a workforce that advances oral health equity [15].

Our findings demonstrate that HUCD has maintained a long‐standing commitment to professional diversity. A substantial body of literature has shown that racial and ethnic concordance between providers and patients improves both access and outcomes [8, 10]. HUCD's success in recruiting and graduating URM students thereby positions the college as a critical force in reducing oral health disparities. Emphasizing this connection places our findings within a broader evidence base on the significance of workforce diversity.

Although our findings highlight the significant role of HUCD in increasing diversity within the dental workforce and addressing geographic imbalances in oral care in the Washington, DC area, these results should be viewed in the context of a single‐institution study. Nationwide, the average percentage of Black professional students enrolled in U.S. dental colleges from Fall 2010 through Fall 2019 was reported to be 6.3%, whereas HUCD reported that 50% of its students identified as Black and African American among classes from 2019 to 2023 [16]. While this difference emphasizes HUCD's notable contribution to training Black dentists, more comparative research across different institutions is necessary to understand how these outcomes relate to broader national patterns.

### 4.1 Regional Distribution and Workforce Impact

Howard University dental graduates, who practice in federally designated DHPSAs and MUAs, are distributed across five national regions, predominantly in the Northeast (354 active dentists) and Southeast (246 active dentists). This regional concentration underscores the historic and ongoing role HUCD plays in shaping the oral health landscape of communities with significant unmet needs. The density of HUCD graduates in these regions is particularly noteworthy given the well‐documented shortage of dental providers in both urban and rural underserved communities [17].

### 4.2 Geospatial Concentrations and Minority Community Service

Geospatial analysis revealed a high concentration of HUCD dental graduates practicing in Washington, DC, Maryland, and Virginia. Outside the primary concentration in the DC, Maryland, Virginia region, smaller clusters of Howard dental graduates appear in New York, Florida, Georgia, Texas, and California. These graduates frequently serve urban communities with greater minority representation, working in areas where African American and Hispanic/Latino populations average 19.98% and 20.10%, respectively, higher than national practice location averages.

Prior studies confirm that URM dentists are more likely to serve patients of their own race and ethnicity, often in communities with limited access to care [18, 19]. This trend of racially concordant care carries important implications: it improves communication, builds patient trust, reduces missed appointments, and enhances treatment compliance. Reflecting this dynamic, more than 50% of HUCD dental graduates identify as URM population, providing clear evidence that HUCD's mission‐driven enrollment strategy translates into a representative workforce that directly impacts underserved populations.

### 4.3 Oral Health Inequities in Washington, DC

An analysis conducted within Washington, DC, using the 2SFCA method, revealed a disproportionate distribution of dentists predominantly in the western regions, while the majority of patients reside in the eastern sectors [20]. Significantly, Wards 5, 7, and 8, areas with the highest proportions of African American residents, exhibit the lowest availability of dental practitioners. Accessibility scores further corroborate this disparity in oral health services. Wards 1, 2, 3, 4, and 6 display relatively high accessibility scores, in contrast to the substantially lower scores observed in Wards 5, 7, and 8. These wards also have the highest concentrations of African American residents, underscoring a critical gap in oral health accessibility in Washington, DC. The findings of this study illustrate that graduates from Howard University's dental program deliver oral healthcare to underserved populations within the DC, Maryland, Virginia region, thereby directly addressing existing disparities.

Within the larger framework of healthcare policy and dental education, these results highlight the ability of institutions to build a workforce that is representative of underserved populations, while producing providers committed to reducing disparities in oral health outcomes. As dental programs across the country consider their aim to broaden their student body, Howard's achievements offer a valuable blueprint in intentional recruitment, comprehensive clinical education, and an ingrained mission of serving marginalized and under‐resourced populations.

### 4.4 Broader Implications for Dental Education and Policy

These outcomes underscore how dental education can be intentionally structured to yield equity‐driven workforce outcomes. HUCD's example demonstrates that targeted recruitment, pathway programs, and mission‐driven curricula can effectively prepare dentists to serve marginalized communities. National reports, such as Advancing Dental Education in the 21st Century [21, 22], call for systemic reforms to broaden student bodies and address oral health inequities. HUCD's long‐standing record provides a blueprint for operationalizing these recommendations, showing that recruitment of URM students, coupled with clinical training that prioritizes underserved populations, can measurably impact community health.

Looking ahead, the future of dental education will require adaptation to new challenges, including integrating emerging technologies like artificial intelligence into training models [23, 24]. HUCD's outcomes suggest that technology‐driven reforms should not come at the expense of equity‐driven missions; rather, they should be pursued in tandem, ensuring that innovations in dental training reinforce rather than dilute commitments to serving vulnerable populations. Expanding the pathway of African American dentists through targeted recruitment, continued support for HBCU dental schools, and workforce policies that incentivize service in shortage areas is an essential component not only for diversifying the profession but also for improving access to care and advancing oral health outcomes nationwide [25].

### 4.5 Study Limitations and Future Directions

This study, while informative, is not without limitations. Publicly sourced data may omit certain graduates and provide an inaccurate classification of dental graduates. Additionally, factors such as geographic relocation and changes in personal identifiers have complicated the process of practice verification.

Future research should address these limitations through longitudinal studies that track HUCD graduates’ contributions over the course of their careers, particularly in the DMV region. Incorporating patient‐level data would provide critical insights into how racial concordance and cultural competence translate into measurable oral health outcomes, such as reduced disease burden or improved preventive care. Additionally, comparative analyses with other dental programs across the United States could identify whether HUCD's impact is unique or reflective of broader national trends, offering opportunities to develop scalable best practices.
